# Facilitators, context of and barriers to acute coronary syndrome care at Kenyatta National Hospital, Nairobi, Kenya: a qualitative analysis 

**DOI:** 10.5830/CVJA-2018-013

**Published:** 2018

**Authors:** Bahiru Ehete, D Huffman Mark, Temu Tecla, Mwanga Julia, Ndede Kevin, Vusha Sophie, Gitura Bernard, Farquhar Carey, Bukachi Frederick

**Affiliations:** Northern Pacific Global Health Research Fellowship Training Consortium, University of Washington, Seattle, WA; and Division of Cardiology, Department of Medicine, David Geffen School of Medicine, University of California, Los Angeles, USA; Department of Preventive Medicine, Northwestern University, Chicago, IL, USA; Department of Global Health, University of Washington, Seattle, WA, USA; Department of Medicine, University of Nairobi, Nairobi, Kenya; Department of Medicine, University of Nairobi, Nairobi, Kenya; Organization International Centre for Reproductive Health Kenya (ICRHK), Kenya; Division of Cardiology, Department of Medicine, Kenyatta National Hospital, Nairobi, Kenya; Departments of Global Health, Epidemiology and Medicine, University of Washington, Seattle, WA, USA; Department of Medical Physiology, University of Nairobi, Nairobi, Kenya

**Keywords:** acute coronary syndrome, sub-Saharan Africa, global health, qualitative research

## Abstract

**Background:**

The prevalence of ischaemic heart disease and its acute manifestation, acute coronary syndrome (ACS), is growing throughout sub-Saharan Africa, including Kenya. To address this increasing problem, we sought to understand the facilitators, context of and barriers to ACS care at Kenyatta National Hospital, with the aim of improving the quality of care of ACS.

**Methods:**

We conducted in-depth interviews with healthcare providers involved in the management of ACS patients from January to February 2017 at Kenyatta National Hospital in Nairobi, Kenya. We selected an initial sample of key participants for interviewing and used a snowballing technique to identify additional participants until we achieved saturation. After transcription of audio recordings of the interviews, two authors conducted data coding and analysis using a framework approach.

**Results:**

We conducted 16 interviews with healthcare providers. Major themes included the need to improve the diagnostic and therapeutic capabilities of the hospital, including increasing the number of ECG machines and access to thrombolytics. Participants highlighted an overall wide availability of other guideline-directed medical therapies, including antiplatelets, beta-blockers, statins, anticoagulants and ACE inhibitors. All participants also stated the need for and openness to accepting future interventions for improvement of quality of care, including checklists and audits to improve ACS care at Kenyatta National Hospital.

**Conclusion:**

Major barriers to ACS care at Kenyatta National Hospital include inadequate diagnostic and therapeutic capabilities, lack of hospital-wide ACS guidelines, undertraining of healthcare providers and delayed presentation of patients seeking care. We also identified potential targets, including checklists and audits for future improvements in quality of care from the perspective of healthcare providers.

Sub-Saharan African countries, including Kenya, are experiencing a rapid rise in the prevalence of ischaemic heart disease and its risk factors, including aging, hypertension, diabetes, obesity, physical inactivity and dyslipidaemia in the context of urbanisation and globalisation. The need to strengthen the health system in sub-Saharan Africa to adequately respond to the growing trends of non-communicable chronic diseases (NCDs), including ischaemic heart disease, is recognised by the World Health Organisation (WHO).^1^ Local cardiology societies such as the Pan-African Society of Cardiology (PASCAR) and the Kenyan Cardiac Society (KCS) advocate and support efforts to increase understanding of the burden of ischaemic heart disease and its acute manifestations such as acute coronary syndrome (ACS) in this region, with the goal of building upon and improving current management trends.^2^

To increase the understanding of ACS care in Kenya, our team conducted a retrospective evaluation of the presentation, management and outcomes of ACS patients managed at Kenyatta National Hospital between 2013 and 2016.^3^ The study has helped to describe current ACS management trends to identify important areas for future improvement of quality of care. To complement our quantitative evaluation of current ACS care at Kenyatta National Hospital, we also conducted a prospective qualitative analysis to understand facilitators of, barriers to and the context of in-hospital ACS care. We sought to identify knowledge, attitude and behaviour about interventions for improvement of quality of healthcare through in-depth interviews with healthcare providers involved in the management of ACS patients at Kenyatta National Hospital. This qualitative evaluation will provide informative data for future activities to improve quality of care in the hospital and region.

## Methods

This qualitative study included in-depth interviews of key participants who were healthcare providers involved in the management of ACS patients at Kenyatta National Hospital, which is one of the two main public referral centres in Kenya. The hospital has Kenya’s most advanced diagnostic and management capabilities for ACS care, including having the only public cardiac catheterisation laboratory in the country.

We developed interview guides to explore facilitators of, barriers to and context of in-hospital ACS care at Kenyatta National Hospital. We modelled this qualitative study based on our team’s prior research in India, which has led to the development of a theoretical model that viewed ACS care through a patient-orientated process map including five stages: (1) prior to first medical contact, (2) at the point of first medical contact, (3) early hospitalisation, (4) mid-to-late hospitalisation, and (5) at the point of discharge.^4^

Starting in January 2017, we selected an initial sample of hospital leaders for interviewing and used a snowballing technique to identify additional participants during February 2017. We used the principle of maximal variability sampling to seek new participants to achieve a diverse sample. We continued our recruitment until we achieved saturation of major themes identified during our analysis.

All interviews with audio recordings were conducted by one interviewer (EB) in English and lasted between 36 and 65 minutes. Audio transcripts and interview field notes of the first three transcripts were independently coded by two individuals (EB, SV) to develop a comprehensive codebook. The same coders used Dedoose version 7.5.275 to code the remaining transcripts and field notes using the codebook. We also developed and implemented a brief survey to capture demographic data and open-ended responses regarding facilitators of, barriers to and context of ACS care, which were further explored in the in-depth interviews.

The study was approved by the University of Washington institutional review board, the Kenyatta National Hospital/ University of Nairobi ethics and research committee and Northwestern University institutional review board. Written informed consent was obtained from all participants.

## Results

We conducted 16 interviews during the study period, including with one cardiologist, two accident and emergency (A&E) attending physicians, two medical officers in the casualty department, three A&E nurses, and eight medical registrars (Table 1). More than half (56%) of the interviewees were women. We also provide a summary of the major facilitators of and barriers to ACS care at Kenyatta National Hospital that were highlighted by most participants in Tables 2 and 3, respectively.

**Table 1:** Participants’ characteristics

*Participants*	*Number = 16 (%)*
Type of ACS provider	
Cardiologist	1 (6)
A&E room attendants	2 (13)
A&E room medical officers	2 (13)
Nurses	3 (19)
Medical residents	8 (50)
Female	9 (56)

**Table 2:** Facilitators of in-hospital ACS management at Kenyatta National Hospital

*Hospital level*	*Provider level*
The hospital is one of a few institutions that has diagnostics including ECG and echocardiography, and is the only public hospital with a cardiac catheterisation laboratory, although availability of some of these diagnostic services are limited and could be improved Guideline-directed in-patient and discharge. Medical therapy, specifically antiplatelet agents, beta-blockers, statins, anticoagulants and ACE inhibitors are largely available Hospital-fee waiver for certain services is available for patients who are unable to afford emergency medical treatment The hospital has critical care units, both in the casualty department and medical wards, to take care of critically ill patients, including ACS patients Structured follow-up mechanism post discharge through the cardiology clinic Continuing medical education programmes that cover current ACS treatment guidelines	Availability of expert staff including cardiologists, well-trained critical-care nursing staff and medical registrars Well-trained echocardiography technicians Providers that participated in this qualitative research displayed great interest in improving existing ACS care, including potential quality improvement

**Table 3:** Barriers to in-hospital acute ACS management at Kenyatta National Hospital

*Hospital level*	*Provider level*	*Patient level*
No standardised triaging system for patients with chest pain or suspected ACS Inadequate number of ECG machines or lack of routine maintenance if malfunctioning Occasional inadequate availability of essential ACS diagnostic tests, such as cardiac biomarkers Lack of availability of some medicines such as nitroglycerine Thrombolytics are not consistently stocked or are not available most of the time There is a lack of standardised protocol or hospital guidelines for ACS management There is no dedicated coronary care unit and very limited availability of ICU beds and resuscitation rooms. Cardiac catheterisation laboratory is available but currently no primary PCI service No hospital-organised specific training for ACS or other cardiac emergencies	Low level of training on the management of ACS Inadequate number of staff with high patient-to-nurse ratio, especially in the medical wards	Low level of knowledge about symptoms of ACS Inability to afford medical treatment Self-medication using over-the-counter medications Language barriers

PCI, percutaneous coronary intervention.

**Theme 1: There is a significant delay from onset of patient symptoms to presentation at Kenyatta National Hospital**

All participants explained that there is a significant delay from symptom onset to presentation at Kenyatta National Hospital, which seems largely driven by a lack of patient understanding of ACS symptoms that warrant emergent medical attention. This delay is further exacerbated by the inter-hospital transfer system from district hospitals to Kenyatta National Hospital.

‘Of course, from the patient side, delay is a big problem and therefore once they come late we end up doing heart-failure management post MI, many of our people do not have the knowledge that if I have a chest pain, I need to rush to hospital…So, knowledge in our community is an area that we need to educate the community about chest pain’

Other respondents described patients seeking care at pharmacies rather than hospitals, for initial management.

‘Significant delay in presentation from symptom onset because most of the time most Kenyans usually try and buy over-the-counter medications and don’t present unless the pain is severe.’

Inter-hospital transfer delays are also driven by delays in diagnosis at district-level hospitals as well as limited access to ambulances for rapid transport.

‘Our hospital, of course, is a national hospital, so many times we get patients who have been referred and therefore they would have passed one or two other hospitals, being managed for either pneumonia or for an abdominal problem, so they tend to come late.’

**Theme 2: Diagnostic, management and treatment capabilities of the hospital are sub-optimal**

Theme 2.1: Availability of electrocardiogram (ECG) and cardiac biomarkers

Respondents all agreed that the limited availability of functioning ECG machines in the hospital creates a significant barrier to rapid ACS diagnosis. Some patients are referred from the A&E department to the out-patient cardiology department for acute ECG monitoring, whereas other patients do not receive an ECG during their hospitalisation. This assessment parallels findings from our study that retrospectively evaluated current ACS management trends at Kenyatta National Hospital, which showed the rate of ECG acquisition within 24 hours of presentation among non-transferred cases was 71%; a small minority (5%) of patients admitted and managed for ACS did not get an ECG during their entire hospitalisation.3

‘Right now, we are not able to do ECG for our patients because our ECG machine broke down a few months ago. We are in the process of getting one….at the moment what we are doing, we are sending the patients to the cardiology unit…to get their ECG done.’

Participants reported that cardiac biomarkers are generally but not always available.

‘At times, also if the reagents for…the cardiac enzymes, troponin, if they don’t have it in the lab that is a challenge. We usually send, take the samples outside, we tell the relatives to take it outside.’

Theme 2.2: Availability of reperfusion therapy

While Kenyatta National Hospital has a cardiac catheterisation laboratory, primary percutaneous coronary intervention is not available at all times. In-hospital cardiac catheterisation is also not part of the routine management of ACS patients admitted to the hospital, often because of late patient presentation for ST-segment elevation myocardial infarction.

‘The hospital has (a) catheterisation lab but again we are not doing primary PCI at the moment…so most of the patients are being managed medically.’ ‘Why are they being managed medically?’ ‘Cause (sic) many times they will come late.’

All participants mentioned that thrombolysis for reperfusion therapy for eligible ACS patients is currently not available at the hospital and attributed cost as the primary reason the hospital does not consistently stock thrombolysis medications. However, the lack of in-hospital thrombolytic availability further limits its use.

‘Currently this hospital does not stock [thrombolysis medicines] in the hospital and therefore even if these patients came on time and could benefit from lysis the relatives have to be given the prescription to go out there and purchase this medicine. So, you can imagine by the time all that is done, the patient came late, by the time the relatives go and buy these medicine, it is never going to be on time.’

Respondents also acknowledged that many providers do not have adequate training to administer thrombolytic therapy.

‘…if we were to get a patient who comes on time and residents make that decision to thrombolyse this patient, are they comfortable with the thrombolysis?’ ‘I would say no.’

Cost of cardiac catheterisation and intervention is another significant barrier. Patients or their relatives need to purchase coronary stents, which is not feasible during the acute treatment period.

‘Of course, the patients have to pay for this angiogram … it is always economically easier for the relatives if [the patients] are discharged through the cardiology clinic, they come and book for that angiogram, and they source for the money…. So, we do tend to do angiogram usually in the course of one week to a month.’

**Theme 3: Guideline-directed in-hospital and discharge medical therapy such as antiplatelets, beta-blockers, statins and anticoagulants are largely available**

Participants reported that other guideline-directed medicines recommended by the current international ACS guidelines such as the American College of Cardiology/American Heart Association or the European Society of Cardiology are available at Kenyatta National Hospital, including aspirin, clopidogrel, nitrates, beta-blockers, statins, ACE inhibitors, oxygen, morphine and anticoagulants. Stock-outs are rare.

‘We have definitely the oxygen points and the oxygen supplies. We have the analgesics, the operators are there. Nitroglycerin would be there. Aspirin, clopidogrel would be there. If maybe (there) was hypertensive emergency, the drugs would be there. There would be the beta-blockers, and ACE inhibitors are available.’

Similarly, guideline-directed medicines are available for prescription at the time of discharge.

‘…of course they will be discharged with all the drugs which are useful for coronary artery disease; then they will be followed up in the cardiology clinic.’

However, participants acknowledged that even if these medicines are available, many patients have limited or delayed access, largely due to cost, which influences long-term adherence. Patients may have to pay for these medications prior to administration, especially in the casualty department.

‘The bad thing is that they have to dig deep into their pockets to take care of [treatment]… the good thing also is that the hospital allows us, especially in emergency set up, allows us to waive all the cost.’

**Theme 4: Lack of awareness and use of standardised hospital protocol to guide ACS management**

Most participants stated that they were not aware whether there is a standardised hospital-wide ACS protocol to guide management of patients. Two participants acknowledged that there is a guideline for ACS management in the casualty department, drafted by the emergency medical services of Kenya, although it is currently not widely distributed to healthcare providers.

‘We don’t have any pinned-up protocols on the wall yet, but there are some protocols we are using from the emergency medical services of Kenya. We are able to disseminate them to the doctors, right now we are in the process of printing, we want to make them into small notebooks and give the nurses and the doctors.’

However, awareness and use of any existing ACS protocol was not universal.

‘We don’t have (a) protocol. When you get in, you do what you see everyone else do.’

**Theme 5: Most staff feel inexperienced managing ACS patients**

Most participants highlighted that they generally feel inexperienced in managing patients with ACS. Participants stated most of their knowledge on management of ACS cases came from self-initiated review of international guidelines,^6^ peers or experiences from working in other institutions.

‘We follow guidelines, most of them are British or American guidelines. European Society for Cardiology, American Cardiac Society, but we only do what is available. And also, sometimes the norms if you are a new resident, like when I started working here, you find what other residents have been doing. So that is what you do, or when you get an ACS patient you consult, you have a resident who can tell you this is usually done.’

**Theme 6: Acceptability of interventions for improvement of quality, including checklists, audits and feedback reports**

All participants made several suggestions on how to improve existing ACS care at Kenyatta National Hospital. Table 4 summarises these recommendations, which are primarily targeted at hospitallevel infrastructure, provider-level ACS training, and community level awareness of ACS management. At the hospital level, recommendations focused on increasing current diagnostic and therapeutic capabilities, such as ECG machines and thrombolytics and implementing a hospital-wide standardised ACS protocol. At the provider level, the most common recommendation focused on improving current training on ACS management.

**Table 4:** Participants’ suggestions for future improvement in quality of ACS care

*Hospital level*	*Provider level*	*Patient level*
Increase diagnostic capabilities, primarily increased number of ECGs in the hospital Have a dedicated ECG machine at triaging point in the accident and emergency room Ensure consistent availability of thrombolysis medicines Improve other laboratory capabilities, such as point-of-care cardiac markers Implement a standardised protocol or hospital guidelines for chest pain triaging and ACS management Build a dedicated coronary care unit	Improve knowledge of healthcare providers on ACS management guidelines Training and protocol on safe administration of thrombolytics Hospital-sponsored advanced cardiac life-support training	Public health initiative to improve patient knowledge on recognition of ACS symptoms and need for emergent medical evaluation Evaluate mechanisms to cover medical costs for ACS care, including expansion of the national health insurance fund to cover essential treatments

We also assessed the acceptability of initiatives to improve quality of care of ACS, such as checklists, audits and feedback reports, which have been shown to improve processes of care in ACS management.^7^,^8^

‘A checklist would definitely be very useful because a lot of the times as residents we manage all sorts of different case presentations... So I think having a checklist just reminds you that there might be an important step that I skipped so you can easily go back to it before it’s too late.’

Most participants described the use of other checklists at the hospital for intensive care unit (ICU), tuberculosis (TB) and trauma services.

‘ICU and TB and chest wards have existing checklists that are standard across public hospitals, especially the TB checklist that has standardised the care for TB patients in the hospital. If there is a specific checklist for ACS patients, that could improve the care.’

## Discussion

This qualitative research study describes facilitators of, barriers to and context of ACS care at Kenyatta National Hospital. The most prominent facilitators mentioned by the majority of participants highlighted that Kenyatta National Hospital is one of two main public referral and teaching centres with the highest capability for ACS diagnostics and therapeutics, including the only public hospital with a cardiac catheterisation laboratory, expert consultants such as cardiologists, and cardiac surgery.

However, all participants highlighted that there are several facility-, provider- and patient-level barriers to optimal ACS management. At the facility level, sub-optimal diagnostic capabilities, especially the very limited number of ECG machines in the hospital, was listed as one of the most significant barriers to making prompt diagnosis when ACS is suspected. A limited supply of thrombolysis medications and adaptation of standardised ACS protocols were listed as additional barriers.

All participants had positive attitudes towards both checklists and audit and feedback systems as key tools to improve ACS care. Some participants described existing checklists such as ICU, tuberculosis or trauma care checklists as examples that a toolkit for improvement of quality of ACS care that included checklists could be feasibly incorporated at the hospital.

We know of no other studies from sub-Saharan Africa that have evaluated facilitators of, barriers to and context of ACS care, using qualitative research methods. There are similar studies from high-income and other low- and middle-income countries that have used qualitative research as a tool to guide future targets and tailor solutions to improvement of quality of care. For example, a 2001 qualitative study at eight US hospitals explored initiatives, strategies and approaches to improvement of care for patients with acute myocardial infarction. This study showed that shared goals for improvement, substantial administrative support, strong physician leadership, and use of credible feedback data were mechanisms used in hospitals that improved their processes of care, such as medication use, compared to those that did not.^9^

Themes from a 2010 study in Egypt, which assessed barriers and opportunities to implement an ACS registry included the need to build a culture of applied research, the importance of modelling a blame-free culture, and the potential of clinical registries as cost-effective investments to support improvement in quality of care for ACS in low- and middle-income countries. Limited human resources and technical infrastructure were two key constraints identified.^10^

A 2016 qualitative study from Kerala, India, evaluating pre-hospital ACS care has been useful in identifying areas for improvement of quality in pre-hospital ACS care.^4^ The study found lack of recognition of ACS symptoms that warrant emergent evaluation, high cost of ACS treatment, specifically cardiac catheterisation, insufficient transport systems, and infrequent use of medical emergency services by the public as contributors to pre-hospital delays.^4^

The framework of the WHO’s health system building blocks consists of service delivery, health workforce, health information systems, access to essential medicines, financing and leadership as key target components of improving access to quality healthcare.^2^ This framework can be used to place our results into context. For example, service delivery requires accurate diagnosis for appropriate management, which highlights the primacy of functioning ECG machines for improving ACS care. The WHO includes ECGs as an essential diagnostic technology in its package of essential non-communicable disease interventions (PEN), and therefore they should be a priority for improving ACS care.^11^

Another example is the need for a health information system to build the evidence base and plan for appropriate and timely allocation of healthcare providers and treatment. An audit and feedback system for ACS and other acute cardiovascular conditions would be one potential mechanism to strengthen the Kenyan health system for better ACS performance and outcomes. The WHO also recommends a comprehensive human resources information system to monitor the health workforce to assess needs and guide appropriate training and utilisation of the health workforce.

Notably, these interviews were conducted during a period when there was a nationwide physician strike in Kenya that lasted 100 days and affected public institutions, including Kenyatta National Hospital.^12^ A physician strike shows the potential fragility of low- and middle-income country health systems and the challenges in improving quality and safety.

In terms of financing, Kenya spends 6.4% of its gross domestic product on healthcare, which is relatively low compared with global peers.^13^ However, 40% of this spending comes from government sources. Future expansion of Kenya’s healthcare expenditures, particularly in the context of achieving universal access to healthcare, will need to account for the growing disease burden of ischaemic heart disease, its acute manifestations such as ACS, and underlying risk factors, to create a sustainable, responsive, high-quality health system that offers financial protection to its citizens.

Our study has some limitations, including being a single location at a public referral hospital; however, ours is the first study of its kind in the region. Another major limitation of this study is that we did not include patients among those interviewed, which is an area of future research for our team. We plan to explore patients’ perspectives in areas of pre-hospital delay, patients’ knowledge about ACS, experiences in receiving ACS-related care and patients’ medical costs.

## Conclusions

This qualitative research assessed facilitators of, barriers to and context of ACS care at Kenyatta National Hospital. These results provide the novel perspectives of healthcare providers on the current trends of ACS management, and potential areas of limitations and opportunities to improve ACS care and outcomes.
